# Epigenetic Memory as a Basis for Intelligent Behavior in Clonal Plants

**DOI:** 10.3389/fpls.2016.01354

**Published:** 2016-08-31

**Authors:** Vít Latzel, Alejandra P. Rendina González, Jonathan Rosenthal

**Affiliations:** ^1^Institute of Botany of Czech Academy of SciencesPrůhonice, Czech Republic; ^2^Ecological Research Institute, KingstonNY, USA

**Keywords:** asexual reproduction, DNA methylation, plant memory, between ramets communication, 5-azacytidine, zebularine

## Abstract

Environmentally induced epigenetic change enables plants to remember past environmental interactions. If this memory capability is exploited to prepare plants for future challenges, it can provide a basis for highly sophisticated behavior, considered intelligent by some. Against the backdrop of an overview of plant intelligence, we hypothesize: (1) that the capability of plants to engage in such intelligent behavior increases with the additional level of complexity afforded by clonality, and; (2) that more faithful inheritance of epigenetic information in clonal plants, in conjunction with information exchange and coordination between connected ramets, is likely to enable especially advanced intelligent behavior in this group. We therefore further hypothesize that this behavior provides ecological and evolutionary advantages to clonal plants, possibly explaining, at least in part, their widespread success. Finally, we suggest avenues of inquiry to enable assessing intelligent behavior and the role of epigenetic memory in clonal species.

## Introduction

Although it has historical antecedents in remarks by Darwin (1880), the more recent and not-universally held characterization of plants as intelligent organisms is largely attributable to a series of publications by Trewavas (2003, 2004, 2005, 2014). Indeed, Trewavas’s arguments for plant intelligence, coupled with counterarguments (Firn, 2004) to his viewpoint, capture the overall discussion of this subject quite well. In the present paper, we propose investigation of a system that is likely to especially strongly display those behaviors that Trewavas – and we – would characterize as intelligent; moreover, this system is particularly well-suited to address the arguments put forth to dispute plant intelligence. Finally, we hypothesize that to the extent that intelligence is manifested in the behavior of the focal organisms – clonal plants – it may at least in part explain the ecological and evolutionary success of their life strategy.

First, a brief overview of Trewavas’s arguments and the proffered rebuttals is in order. As he noted (Trewavas, 2003), the traditionally prevailing presumption that plants lack intelligence is likely largely due to methodological difficulties and biases in applying to plants concepts originally developed in the context of animals. In particular, because the traditional, zoocentric measure of behavior has been in terms of movement, plants’ sessile lifestyle and different reaction time-scale has probably helped create the widespread view of plants as passive and therefore incapable of behaving, let alone exhibiting intelligence. Similarly, Trewavas (2003) noted that because many plant responses to environmental conditions are manifested in terms of growth, or through such cellular-level processes as changes in turgor pressure, it is tempting to dismiss these responses as developmental or physiological processes, rather than behavior. However, given their sessile nature, it is through such directed growth that plants can explore and preferentially exploit favorable environments, akin to the movement shown by animals.

Therefore, Trewavas (2003) employed Stenhouse’s (1974) objective definition of intelligence as “adaptively variable behavior within the lifetime of the individual”, because it is not *a priori* inapplicable to plants; however, he did so with the understanding that “behavior” be limited to responses on the level of the whole plant (i.e., excluding direct responses of a single organ or tissue to the environment with which it is interacting). Along with many examples of intelligence so defined, he described (Trewavas, 2003) various plant capabilities that make such intelligence possible, including plant learning abilities (e.g., Shen-Miller, 1973; Ishikawa et al., 1991) and communication and decision-making capabilities (Addicott, 1982; Griffiths and McIntyre, 1993; Takayama and Sakagami, 2002).

Trewavas’s characterization of plants as intelligent was disputed (Firn, 2004) on multiple bases, the most salient of which can be distilled down to the contentions that the concept of the individual is largely inapplicable to plants (as they are not only modular, but organs can exist on their own), that in any case they do not respond as individuals (with responses made instead at the organ level and without coordination), that memory such as it exists in plants is similarly localized (i.e., there is no repository of plant-wide memories analogous to a brain) and simply consists of linear sequences of developmental experiences, and that, unsurprisingly, given these limitations, plants do not make meaningful choices. In turn, Trewavas (2004) rebutted these assertions by, among other things, citing evidence that generally plant parts cannot survive on their own; that responses are coordinated between plant parts (e.g., roots controlling shoot growth or vice versa), relying upon communication between them; that memory – albeit not residing in a central repository – operates at a plant-wide level; and that plant decision-making goes well beyond merely achieving homeostasis or making simple binary choices. Moreover, much more evidence of the complexity and coordination of plant behavior has been adduced over the years since Trewavas’s initial publications.

Of special relevance to the study system that we discuss, Trewavas (2003), rather than seeing modularity as an obstacle to intelligent behavior, suggested that “[a] consequence of a repetitive modular structure is that the individual ramets might be regarded as being like parallel processors contributing different experiences resulting from different ages to present day decisions.” Following up on this idea, we hypothesize that the additional level of modularity displayed by clonal plants that occur as networks of connected individuals could confer a greater capacity for intelligent behavior in comparison to otherwise similar plants that occur as fully separate individuals plants (with the relevant comparisons being between plants of the same growth form and similar size, such that, e.g., clonal herb individuals can be compared to those of non-clonal herbs, clonal shrub individuals to non-clonal shrub individuals and clonal tree individuals to non-clonal trees). The plants in such a network would benefit not only from the varied experiences of the constituent individuals but also from modes of communication and coordination that rely upon physical connections (and which would complement those modes that are also available to fully separate individuals). Additionally, we hypothesize that epigenetic modification, which has already been suggested as a plant memory mechanism (Gagliano et al., 2014; Trewavas, 2014) may be especially important in clonal plants, potentially contributing further to heightened capacity for intelligent behavior. Here we present these hypotheses against an overview of recent advances elucidating plant learning and communication capabilities, and then propose particular research approaches to ascertain whether epigenetic modification does in fact contribute to adaptive behavior in clonal plants. Finally, we briefly explore the possible role of the potential, associated fitness advantage of heightened intelligence in the proliferation of clonality in plants.

## Remembering and Learning From Environmental Interactions

There have been ample demonstrations of plant abilities to not only store information about their environmental interactions, even for several generations, but to also properly evaluate that information in the context of ambient conditions (Galloway and Etterson, 2007; Sultan et al., 2009; Whittle et al., 2009; Latzel et al., 2010, 2014). Moreover, plants have even been shown to predict future conditions based upon past experiences. Thus, plants can perform quantum computing in chloroplasts to anticipate future light quality changes based on former light quality, enabling them to adjust photosynthetic apparatus accordingly (Karpinski and Szechynska-Hebda, 2010; Szechynska-Hebda et al., 2010). Similarly, plants can also modify their growth in accordance with expected future resource competition based on previous experience (Shemesh et al., 2010; Novoplansky, 2016). Finally, memories of previous environmental stress can also help plants better overcome future stress (Ding et al., 2012).

Learning from past experiences requires memory. Although some memory mechanisms such as accumulation or stable modification of key regulatory proteins cannot be separated from developmental processes, most plant memories are likely enabled by molecular mechanisms, of which epigenetic modification is probably the most prominent (Bruce et al., 2007; Ginsburg and Jablonka, 2009; Ding et al., 2012; Thellier and Lüttge, 2013; Müller-Xing et al., 2014). Epigenetic mechanisms such as histone modifications and reversible methylation of cytosine alter gene expression and thus also phenotypes (Liu and Wendel, 2003; Bird, 2007). Because epigenetic variation is environmentally inducible and mediates phenotypic plasticity (Jablonka and Lamb, 1995; Alvarez et al., 2010), it is very likely that many of the environmental interactions of an individual are reflected in epigenetic change.

Environmentally induced epigenetic change can become fixed for a period (Chinnusamy and Zhu, 2009) and eventually may become heritable, thus altering plant-environment interactions in subsequent generations (Molinier et al., 2006; Boyko et al., 2010; Verhoeven et al., 2010). Epigenetic variation can thus enable plants to remember their former interactions with the environment, learn from them, and apply the acquired knowledge to improve their performance in the future (e.g., Boyko et al., 2010; Kou et al., 2011; Bilichak et al., 2012; Ding et al., 2012; Müller-Xing et al., 2014). Moreover, such behavior, because it can be performed at any stage of plant development, is clearly independent of developmental progression, and thus overcomes the objection that supposed behavior is actually just development and therefore not a manifestation of intelligence.

## The Potential for Clonality and Epigenetics to Combine in Facilitating Plant Intelligence

Clonal reproduction can yield genets comprising up to several hundred connected ramets (Kemperman and Barnes, 1976). Each ramet essentially constitutes an individual plant, generally capable of persisting independently if it becomes disconnected from the rest of the genet spontaneously or by injury. However, the interconnected genet functions in some ways as a single entity, with stressed ramets able to avert death by drawing on the support of stronger ramets (Fischer and Stöcklin, 1997), labour division among ramets (Alpert and Stuefer, 1997), and also with the overall growth of a genet (Stuefer and Hutchings, 1994) increased by its capability to share resources among ramets.

Genets also have the ability to adjust to resource heterogeneity by placing ramets in favorable patches (Bell, 1984; Waters and Watson, 2015), showing that they take account of spatial environmental variation, which is especially likely to be encountered as they spread. Additionally, because a genet typically produces new, connected ramets over time, such that there is coexistence of ramets of different ages (as mentioned by Trewavas, 2003), the ramets within a genet are likely to have been produced not only over spatially varying environmental conditions, but also over temporally varying conditions. If information on such variation can be stored, shared, and translated into appropriate responses, then this can form the basis for particularly intelligent behavior on the part of clonal plants.

Clonal plants do appear to have greater ability than non-clonal plants to remember past environmental interactions (Jablonka and Lamb, 2005; Latzel and Klimešová, 2010; Verhoeven and Preite, 2014). This may be due at least in part because of their more effective transfer of epigenetic information among clonal generations (Jablonka and Lamb, 2005; Latzel and Klimešová, 2010; Verhoeven and Preite, 2014), in contrast to the resetting of most environmentally induced epigenetic information during meiosis during sexual reproduction (reviewed by Paszkowski and Grossniklaus, 2011). In fact, the more faithful intergenerational transmission of epigenetic information would provide an advantage to ramets (relative to sexually produced individuals) even if they become detached from their parent clone and are living independently.

Nevertheless, it is within connected genets that epigenetic memory is likely to have its greatest effect in terms of facilitating intelligent behavior of clonal plants. This is because although even populations of non-clonal plants can communicate via root exudates or volatiles (e.g., Falik et al., 2011), the physical connections maintained within such genets allow communication by additional means. Communication within interconnected ramets has been demonstrated (Stuefer et al., 2004), particularly with regard to early warning systems in which information of an herbivore attack can be spread throughout the clone, enabling those ramets not yet attacked to produce defenses (Gómez et al., 2007). Hormones such as jasmonic and salicylic acids are thought to carry such signals within a clonal network. Although they can provide information advantage for clonal plants, these messengers would carry information only about the situation currently or very recently encountered by the genet, and would not convey information about events or conditions from the more distant past.

Based on studies by Raj et al. (2011) and Richards et al. (2012) showing that clonal, genetically homogeneous populations of *Populus* and *Fallopia*, respectively, remember former environments via epigenetic variation, we propose that environmentally induced epigenetic change might be the mechanism that can carry precise information about environmental interactions from the more distant past. We suggest that given the absence of meiotic resetting of epigenetic modification in clonal reproduction, newly emerging offspring ramets can inherit epigenetic information of previous environmental interactions from the maternal ramet. Moreover, because an offspring ramet can encounter different environments than those experienced by the maternal ramet, the epigenetic information that will ultimately be conveyed by the offspring ramet to the generation subsequent to it (i.e., third generation) can represent a combination of maternal (first generation) and offspring (second generation) environmental interactions.

Indeed, from studies on *Arabidopsis thaliana*, we know that a single genotype can have more than 250 distinct stable epigenetic states (Johannes et al., 2009; Cortijo et al., 2014). Thus, in a genet consisting of tens or hundreds of interconnected ramets, each ramet is likely to have a different epigenetic state depending not only upon the environmental conditions experienced by itself and its progenitors but also upon the interactions between these conditions and the phenotypes in each of these generations. This variation among connected individuals, each of which consists of a whole, potentially independent plant, in combination with the enhanced capability for communication among them, would, we suggest, provide the basis for “swarm intelligence”, an emergent property of cooperating groups that enables them to solve problems that are beyond the abilities of a single member (Bonabeau et al., 1999). Indeed, in seeking to explain the decentralized intelligence exhibited by individual plants, Trewavas (2005) suggested looking at “other decentralized intelligence systems such as those found in social insects.” This analogy is even more appropriate when considering the potential swarm intelligence of ramets within a connected genet.

Although we are primarily concerned with the relative capacity for intelligent behavior of a connected genet vs an array of non-clonal, yet comparable individuals, the ability for constituent ramets to process information and act somewhat autonomously does raise the question (with possible evolutionary implications) of whether a genet would have an intelligence advantage over a single plant of similar size to the entire genet. In more fully considering this possibility, which is beyond the scope of this paper, an important issue would be whether the possible communications advantages enabled by greater physical integration within a single, large individual would offset the benefit of having a “swarm” of largely autonomous ramets.

One possible product of epigenetically mediated intelligence in clonal plants is pre-programming of offspring ramet phenotypes for particular types of growth based on the previous interactions of the genet with the environment. This can significantly increase overall success of the genet, particularly in cases when environmental conditions are predictable. For instance, adaptive responses to drought may encompass increased investment in roots to better tap ground water and/or reduced leaf growth to reduce water loss (Kozlowski and Pallardy, 2002; Chaves et al., 2003). In the case of simple phenotypic plasticity, the newly emerging offspring ramet would respond to the ambient water level and adjust its growth accordingly. Thus, in the scenario in which rainfall temporarily moistens soil of an otherwise dry environment, a newly emerging ramet would adjust its phenotype to wet conditions and grow shallow roots and large leaves, which could easily become inefficient or even costly for the genet when this moisture disappears. However, knowledge previously acquired by the genet could theoretically modify the plastic response of such a new ramet to better take account of long-term environmental conditions. Such intergenerational phenotypic adjustments are well-documented for sexual generations (Galloway and Etterson, 2007; Sultan et al., 2009; Latzel et al., 2010, Latzel et al., 2014), and we propose that similar effects are likely to occur in clonal plants. Of course, it is possible that constraints imposed on plastic responses to contemporaneous conditions could be detrimental if they reflect conditions that are no longer at all relevant. Nevertheless, we suggest that, the ongoing communication among ramets from differing periods may allow, via swarm intelligence, the genet to evaluate the suitability of differing responses and then, translate this, through signaling and resource allocation, into decisions to favorable outcomes for constituent ramets. Evolutionarily, such intelligent behavior may actually enable maintenance of high phenotypic plasticity of clonal species because educated control of offspring ramet phenotypes by reducing risks associated with high phenotypic plasticity, as described above.

Indirect support for this idea may have been provided by Louapre et al. (2012), who in an experiment on *Potentilla* and *P. anserina* found that as a stolon lengthened, the decision about where to place successive rooting ramets depended not only on resource richness experienced by the newest ramet but also by those preceding it. Additionally, the placement of new *P. reptans* ramets was influenced by the variability in richness among the rooting sites of previous ramets. Thus, for this species, their model system demonstrated that not only the average amount of resources obtained by older ramets, but also the variability of resources among ramets can alter the growing strategy of a whole genet (Louapre et al., 2012). Although they considered only plant hormones or resource molecules as potential messengers in their system, epigenetic variation could also underlie the behavior of the clones in their study.

Variation among epigenetic memories of interconnected ramets can also modify the information values of signals that are sent within the genet. Because epigenetic variation alters plant responses to hormones and other messengers (Latzel et al., 2012), potentially each ramet in a genet could respond differently to signals received. It is also plausible that epigenetically distinct ramets can differently modulate the intensity of signals that they are sending. Thus, the interaction of interconnected ramets can form the basis for a unique system in which the information spread is evaluated and modulated on the basis of epigenetic memory.

For all of the abovementioned reasons, we suggest that epigenetic memory within connected genets can enable information storage and evaluation such that it can provide the basis for variability in decision-making that would overcome objections (e.g., Firn, 2004) that plant decision-making is trivial, thus not manifesting intelligence. Moreover, such variability could enable genetically and even developmentally identical genets to react differently to environmental stimuli such as attack by insect or microbial infection.

## Assessing Intelligent Behavior of Clonal Plants

Although we do not propose a test to quantify the overall intelligence of clonal plants, i.e., something akin to an IQ test, we believe that the extent to which information is stored epigenetically, transmitted across space and time within connected genets, and ultimately incorporated into the decision making of constituent ramets should all be able to be assessed. Thus, conceptually, to test whether epigenetically conveyed information from the past is useful to the plants in making better decisions (i.e., that will yield greater fitness or a proxy thereof), we can compare two genetically identical and developmentally equivalent genets, one naïve, i.e., without such information and one possessing such information about past environmental interactions. If the plants’ behavior were guided only by presently sensed information, without input from acquired memory, then both genets should react similarly to present environmental stimuli. However, if the plants have the ability to evaluate new, ambient signals against a backdrop of stored environmental information, shared through clonal network connections, the genet with relevant experience should behave differently, giving it a considerable advantage over the naïve one.

To specifically examine the role of epigenetic modification in furnishing such memory for decision-making by clonal plants, perhaps the most straightforward way to make this comparison would be to use a demethylating agent that would disrupt already-acquired epigenetic modification. Using this approach, equivalent genets would be exposed to the same sequence of temporally varied environmental conditions, but for one set, their epigenetic memory would be disrupted continuously via DNA demethylation using a demethylating agent such as 5-azacytidine or zebularine. Demethylation has been successfully applied in studies testing the role of epigenetic variation in phenotypic plasticity or adaptation to changed environments (e.g., Tatra et al., 2000; Boyko et al., 2010). These studies germinated seeds in a 5-azacytidine solution, an approach inapplicable to mature clonal plants. However, we have developed a new approach in which mature clonal plants are sprayed repeatedly with an aqueous solution containing 5-azacytidine and surfactant for months. Azacytidine is absorbed by leaves similarly to nutrients from a foliar fertilizer. Because of the constant exposure of growing plants to the demethylating agent, the newly created offspring ramets have modified methylation patterns. Our first results show that the method can lead to about 4-8% reduction in global methylation level of DNA in *Trifolium repens* (Gonzalez et al., unpublished) and *Taraxacum brevicorniculatum* (Dvořáková et al. unpublished) without significant effects on plant phenotypes. Although this method may have side effects that can create unknown artifacts, such an approach can serve at least as a proof-of-principle, and if the side effects are negligible or can be accounted for, it can yield useful observations.

A more sophisticated (and complicated) approach would be to grow genetically identical, developmentally equivalent genets, and subject them to innocuous variation in environmental conditions that could serve as a signal for subsequent, more consequential environmental changes (i.e., equivalent to such signals used for conditioning in behavioral experiments on animals). By providing such genets with various environmental signals and consequences (e.g., varying herbivory intensity), and then seeing whether past experience favorably alters responses to present conditions, we can assess whether genets are learning from experience and applying this knowledge. The latter approach can be combined with experimental demethylation to determine the mechanistic role of epigenetic modification.

Additionally, to examine the extent to which information is shared and has impacts across a genet, individual ramets can be treated differently (e.g., be subjected to different levels of herbivory) and an assessment can be made of whether and how much their neighbors show responses (over a longer term than would be accounted for by hormones). Similarly, although ramets of different developmental stages may respond differently to particular environmental conditions, effects on neighbors of those at sensitive stages can be assessed, to determine whether a ramet can learn from a connected ramet’s experience. Finally, the extent to which epigenetic variation within a genet contributes to variation among ramets in responses to an environmental stimulus can be assessed by demethylation, which by eliminating some of the epigenetic variation could convert a pattern of highly individualistic responses to one of more uniform responses.

Importantly, all the suggested approaches should try to compare behavior of genets consisting of different number of ramets to test the prediction that intelligent behavior increases with increasing complexity of clonal plants. Additionally, because we propose that intelligence in clonal plants would arise from multiple factors including intergenerational transmission of epigenetic modification and ongoing, enhanced communication through physically connections among ramets with experiences from different times and places, we suggest that research also explicitly examine the role of such communication. Thus, perhaps, comparison can be made between intact clonal networks and networks with connections severed (after the network has already been subjected to varying environments). Or, less intrusively, similar comparisons could be done between networks and equivalent numbers of single ramets of the same species. Additionally, to directly address the question of clonal vs. non-clonal intelligence, the ability of (connected) genets to respond to environmental variation could be compared with that of arrays of similar (and phylogenetically close) non-clonal individuals. Although all of these comparisons would have to overcome challenges, we believe that it is worthwhile to at least suggest such possible avenues of future research.

## Conclusion

Trewavas (2014) suggested that intelligence must be understood in terms of the interaction of the individual with the environment, with the environment posing the problems that intelligence is needed to solve. As examples from the literature show, plants are able to draw lessons from their interactions with the environment and can respond in ways that cannot be characterized as simple physiological or developmental consequences. Moreover, we suggest that clonal networks, especially in conjunction with epigenetic modification, can facilitate intelligent behavior, with epigenetics enabling long-term information storage in response to the environment and the latter permitting not only unaltered transmission of this information to subsequent generations, but also integration of information across multiple units experiencing different conditions.

A longstanding question in plant ecology has been why the clonal lifestyle, despite some likely disadvantages in terms of reduced population genetic variation, is so widespread, and is indeed predominant in some habitats (Klimeš et al., 1997). The posited facilitation of intelligent behavior, if actually present, might at least in part explain the success of clonal species, by enabling them to have more flexible, accurate and efficient responses to varied environments than do non-clonal species.

Not only might clonal plants gain an advantage over non-clonal plants, but it is likely that there are differences among clonal species in their abilities to accumulate and/or employ stored information – i.e., their intelligence. In particular, their intelligence could depend on life-history characteristics such as clonal growth type (e.g., phalanx vs guerrilla strategy), integration level of ramets, level of information exchange among ramets, and persistence of clonal connections.

Results from our first (as yet unpublished) studies on this subject have shown that memories of former environments can persist across several cohorts of ramets and can be erased by demethylation (Rendina González et al., in press). Because the research that our group can conduct is finite and the questions, especially regarding potential advantages that enhanced intelligence could provide to clonal vs. non-clonal plants, would require major undertakings to definitively answer, we hope that this essay will stimulate discussion about our ideas and possibly inspire other researchers to help determine their overall validity.

## Author Contributions

All authors listed, have made substantial, direct and intellectual contribution to the work, and approved it for publication.

## Conflict of Interest Statement

The authors declare that the research was conducted in the absence of any commercial or financial relationships that could be construed as a potential conflict of interest.
